# Complement Regulator FHR-3 Is Elevated either Locally or Systemically in a Selection of Autoimmune Diseases

**DOI:** 10.3389/fimmu.2016.00542

**Published:** 2016-11-28

**Authors:** Nicole Schäfer, Antje Grosche, Joerg Reinders, Stefanie M. Hauck, Richard B. Pouw, Taco W. Kuijpers, Diana Wouters, Boris Ehrenstein, Volker Enzmann, Peter F. Zipfel, Christine Skerka, Diana Pauly

**Affiliations:** ^1^Department of Ophthalmology, University Hospital Regensburg, Regensburg, Germany; ^2^Institute of Human Genetics, University of Regensburg, Regensburg, Germany; ^3^Institute of Functional Genomics, University of Regensburg, Regensburg, Germany; ^4^Research Unit Protein Science, Helmholtz Zentrum München, German Research Center for Environmental Health (GmbH), Neuherberg, Germany; ^5^Department of Immunopathology, Sanquin Research and Landsteiner Laboratory of the Academic Medical Center, University of Amsterdam, Amsterdam, Netherlands; ^6^Department of Pediatric Hematology, Immunology and Infectious Diseases, Academic Medical Center, Emma Children’s Hospital, Amsterdam, Netherlands; ^7^Department of Blood Cell Research, Sanquin Research and Landsteiner Laboratory of the Academic Medical Center, University of Amsterdam, Amsterdam, Netherlands; ^8^Klinik und Poliklinik für Rheumatologie und Klinische Immunologie, Asklepios Klinikum Bad Abbach, Bad Abbach, Germany; ^9^Department of Ophthalmology, Inselspital, University of Bern, Bern, Switzerland; ^10^Department of Infection Biology, Leibniz Institute for Natural Product Research and Infection Biology, Jena, Germany; ^11^Friedrich Schiller University, Jena, Germany

**Keywords:** FHR-3/CFHR3, specific antibody, rheumatic disease, microglia/macrophage, FH competition, immune therapy, retinal degeneration

## Abstract

The human complement factor H-related protein-3 (FHR-3) is a soluble regulator of the complement system. Homozygous *cfhr3/1* deletion is a genetic risk factor for the autoimmune form of atypical hemolytic-uremic syndrome (aHUS), while also found to be protective in age-related macular degeneration (AMD). The precise function of FHR-3 remains to be fully characterized. We generated four mouse monoclonal antibodies (mAbs) for FHR-3 (RETC) without cross-reactivity to the complement factor H (FH)-family. These antibodies detected FHR-3 from human serum with a mean concentration of 1 μg/mL. FHR-3 levels in patients were significantly increased in sera from systemic lupus erythematosus, rheumatoid arthritis, and polymyalgia rheumatica but remained almost unchanged in samples from AMD or aHUS patients. Moreover, by immunostaining of an aged human donor retina, we discovered a local FHR-3 production by microglia/macrophages. The mAb RETC-2 modulated FHR-3 binding to C3b but not the binding of FHR-3 to heparin. Interestingly, FHR-3 competed with FH for binding C3b and the mAb RETC-2 reduced the interaction of FHR-3 and C3b, resulting in increased FH binding. Our results unveil a previously unknown systemic involvement of FHR-3 in rheumatoid diseases and a putative local role of FHR-3 mediated by microglia/macrophages in the damaged retina. We conclude that the local FHR-3/FH equilibrium in AMD is a potential therapeutic target, which can be modulated by our specific mAb RETC-2.

## Introduction

The human complement factor H-related protein 3 (FHR-3) belongs to the complement factor H (FH)-family. This family, consisting of seven proteins [FH, FH-like protein 1, FH-related protein (FHR) 1–5], are secreted plasma proteins and important regulators of the complement system (1).

The five *cfhr* genes are located on chromosome 1q31.3, downstream of the *cfh* gene (2), coding for FH-family members, which share high sequence identities within their short consensus repeat (SCR) domains. FHR-3 is composed of five SCR domains, which display similarities with SCR6–8 (91–62%) and SCR19–20 (64–37%) of FH (1, 3, 4). Indeed, unambiguous identification and modulation of FHR-3 is challenging considering their high protein sequence similarity. Reported normal systemic FHR-3 concentrations ranged between 0.02 and 100 μg/mL (5–7). The molecular function of FHR-3 is only partly clarified and controversially discussed in the literature (1, 8).

The deletion of the genes for *cfhr1* and *cfhr3* are a double-edged sword as it was genetically associated with protection against age-related macular degeneration (AMD) (9–11) and IgA nephropathy (IgAN) (12), or was associated to be a genetic risk factor for atypical hemolytic-uremic syndrome (aHUS) (13, 14) as well as systemic lupus erythematosus (SLE) (15). FHR-3 was also found in middle ear fluid following alternative complement pathway activation due to infections and was associated with pro-inflammatory activity (16).

Diverse local functions of FHR-3 at different injury-associated altered surfaces were studied (17). All FHR proteins bind to C3b, the central protein of the complement C3- and C5-convertases. Three of the five FHR proteins (FHR-1, FHR-3, and FHR-5) compete with FH for binding to C3b. Thereby, on the one hand, they promote alternative complement pathway activation (5, 18). On the other hand, FHR-3, FHR-4, and FHR-5 show a weak cofactor activity in degradation of C3b by factor I resulting in a reduced alternative pathway activity (1, 3).

According to the gene association studies mentioned before, therapeutic inhibition of FHR-3 could be beneficial in AMD or IgAN, while a drug-dependent increase of FHR-3 activity could be a potential strategy for treatment of aHUS and SLE. A published monoclonal antibody (mAb) against FHR-3 is highly specific, but its effect on the function of FHR-3 has not been evaluated (19). We hypothesize that specific anti-FHR-3 mAbs have the potential to clarify and to modulate the function of FHR-3.

Here, we describe novel-specific mAbs of different isotypes against human FHR-3. Using the highly specific mAb RETC-2 for the evaluation of FHR-3 levels in different human serum samples revealed a significantly increased FHR-3 concentration in rheumatoid patient samples. Furthermore, we identified local production of FHR-3 by microglia/macrophages in an aged donor retina with RPE atrophy – the latter being a typical hallmark of dry AMD. Additionally, we demonstrate that FHR-3 mAb RETC-2 reduced binding of FHR-3 to C3b reinforcing the local binding of the FHR-3 competing complement inhibitor FH. Thus, FHR-3-targeting therapeutics may offer an innovative strategy for local immune therapies for AMD and other complement-related diseases.

## Materials and Methods

### Human Material, Animals, and Ethical Statements

Collection of human blood and eye samples were approved by the local ethics committees (serum: University of Regensburg and Friedrich Schiller University Jena, Germany; eyes: University of Bern, Switzerland) and were obtained in accordance with the Declaration of Helsinki. Complement-depleted human sera were purchased from Complement Technology (Tyler, TX, USA). All human serum samples were stored at −80°C. Balb/c and C57/BL6 mice were obtained from Charles River Laboratories (Wilmington, MA, USA). Mice experiments were strictly performed according to the guidelines of replacement, refinement, and reduction of animals in research (20) and approved by the committee on the ethics of animal experiments of the regional agency for animal health *Regierung der Oberpfalz, Veterinärwesen* (TVA 54-2532.4-05/13).

### Proteins and Antibodies

FHR-1, FHR-2, and FHR-5 purified proteins were obtained from Novoprotein (Summit, NJ, USA). Recombinant FHR-4A and FHR-4B were purified as previously described (6). FH and C3b were ordered from Complement Technology (Tyler, TX, USA). Bovine serum albumin (BSA) was purchased from SERVA Electrophoresis GmbH (Heidelberg, Germany). Heparin-biotin was obtained from Sigma-Aldrich (Munich, Germany).

The goat polyclonal antihuman FH antibody was obtained from Quidel (cat. A312, San Diego, CA, USA), the mouse mAb anti-FH was a kind gift of S. Berra (Università degli Studi di Milano, Milano, Italy) (21), and mouse mAb anti-FHR-3.1 and anti-FHR-3.4 were described previously (6). Rabbit anti-Iba1 polyclonal antibody was ordered from Wako Chemicals (cat. 019-19741, Neuss, Germany), and mouse anti-Glutamine Synthetase mAb, clone GS-6, was from Calbiochem/Merck Millipore (cat. MAB302, Darmstadt, Germany). StrepMAB-classic (cat. 2-1507-001) and StrepMAB-classic conjugated to horseradish peroxidase (HRP) (cat. 2-1509-001) were sourced from IBA (Goettingen, Germany). Immunofluorescence secondary antibodies: rabbit anti-goat Alexa Fluor 546-conjugate (cat. A21085), goat anti-rabbit Cy3-conjugate (cat. A10520), and DAPI (4′,6-diamidino-2-phenylindole, dihydrochloride) were purchased from Thermo Fisher Scientific (Braunschweig, Germany); goat anti-mouse CF488A-conjugate antibody (cat. 20018-1) was ordered from Biotium (Hayward, CA, USA). Rabbit anti-goat IgG-HRP (cat. 305-035-003) and goat anti-mouse IgG-constant part of immunoglobulin (Fc)γ-HRP (cat. 115-035-164) were purchased from Jackson ImmunoResearch (West Grove, PA, USA).

The immunogenic peptide within SCR5 (Charité Universitätsmedizin Berlin, Institute of Biochemistry, Laboratory Proteolytic Systems, Berlin, Germany) was conjugated either to BSA for mouse immunization or to keyhole limpet hemocyanin for ELISA screening of positives clones.

### Expression of Recombinant FHR-3

The construct *pCAG-cfhr3* (22), containing the *cfhr3* sequence with a C-terminal Strep-tag II, was transiently inserted into human embryonic kidney cells 293 (HEK293, Life Technologies, Carlsbad, CA, USA) with TransIT-LT1 Transfection Reagent (Mirus, Madison, WI, USA), according to the manufacturer’s protocol. FHR-3 with Strep-tag II was purified from HEK293 supernatant and cell lysate using Strep-Tactin Sepharose columns (IBA, Goettingen, Germany) (23). After gradient elution of FHR-3 using Strep-Tactin Sepharose columns (IBA, Goettingen, Germany), the recombinant protein was concentrated by vacuum centrifugation. Protein purity was detected with Coomassie staining and Western blot.

### Generation of mAbs

Mouse mAbs against human FHR-3 were generated by hybridoma technology (24). Briefly, mice were subcutaneously immunized with 50 μg peptide-BSA in Freund’s adjuvants (Sigma-Aldrich, Munich, Germany). Spleen cells were isolated, fused, and cultivated as described previously (25). Hybridoma supernatants were tested for specific FHR-3-binding by ELISA. Protein-G affinity purified antibodies from hybridoma clones (HiTrap Protein G HP affinity column, GE Healthcare Life Science, Piscataway, NJ, USA) were named as follows (antibody:hybridoma): RETC (REgensburg Therapy Complement)-2:269-5, RETC-3:353-1, RETC-5:552-3, and RETC-7:773-17.

### Determination of Antibody Variable Regions

RNA of hybridoma cell lines was isolated using RNeasy Mini Kit (Qiagen, Hamburg, Germany) and subsequent synthesis of cDNA using the Quantitect reverse Transcription Kit (Qiagen, Hamburg, Germany). PCR for amplification of IgG variable regions (heavy and light chain) was performed using the mouse IgG Library Primer set (Progen, Heidelberg, Germany), according to the manufacturer’s protocol and DNA-fragments were ligated into pGEM-T Easy plasmid (Promega, Mannheim, Germany) for *E. coli* XL-1 Blue competent cells (Agilent Technologies, Boeblingen, Germany) transformation. Following plasmid isolation (Plasmid Midi Kit, Qiagen, Hamburg, Germany), DNA-sequencing was performed by GeneArt (Thermo Fisher Scientific, Braunschweig, Germany) with pGEM-T specific primer M13 (Promega, Mannheim, Germany).

### Indirect ELISA for Antibody Analyses

PolySorp microtiter plates (Nalgene Nunc, Penfield, NY, USA) were coated with antigen (5 μg/mL, PBS, overnight, 4°C). Each incubation step was finalized with three consecutive washing steps (PBS-T, PBS, 0.1% Tween 20). Blocking was performed with 2% skim milk in PBS/T (1 h). After incubation with anti-FHR-3 mAbs (2% skim milk in PBS-T, 1 h), detection followed by goat anti-mouse IgG-Fcγ-HRP (1:2500, 2% skim milk in PBS-T, 30 min) and 3,3′,5,5′-tetramethylbenzidine (TMB, Seramun Diagnostica GmbH, Heidesee/Wolzig, Germany). Optical density (absorption) was determined at 450 nm.

### Immunoprecipitation of FHR-3 from Human Serum

Five milligrams of tosylactivated dynabeads (Life Technologies, Carlsbad, CA, USA) were either conjugated to 100 μg mAb RETC-2, mAb RETC-3, or the respective IgG isotype controls according to the manufacturer’s protocol. Pooled normal human serum (NHS, 1.5 mL) was incubated with mAb-coupled dynabeads (50 μL, 1 h). After washing, proteins were eluted using non-reducing Laemmli sample buffer (10 min, 95°C) (26).

### Gel and Non-Gel LC-MS/MS Analyses

Samples were separated by SDS-PAGE and stained with Coomassie. Afterward, all visible bands were excised and subjected to in-gel-digestion as published previously (27). Resulting peptides were used for nano-LC-MS/MS analysis on a TripleTOF 5600+ mass spectrometer (Sciex, Darmstadt, Germany) as published previously (24). Database searches were accomplished using the ProteinPilot 4.5 software using the Uniprot-database (version 05/2014).

Samples for non-gel LC-MS/MS were diluted in 50 mM ammoniumcarbonate, then reduced with 100 mM dithiothreitol for 30 min at 60°C, and alkylated with 300 mM iodoacetamide at room temperature. Trypsin-mediated proteolysis was performed using a modified Filter-Aided Sample Preparation protocol as published previously (28). Resulting peptides were used for analysis on a LTQ-Orbitrap XL (Thermo Fisher Scientific, Braunschweig, Germany) connected with an Ultimate 3000 nano-HPLC system (Thermo Fisher Scientific, Braunschweig, Germany) as described previously (29). Peptides were identified and quantified using the Progenesis QI software (Non-linear, Waters) and the Mascot search algorithm with the Ensembl Human public database (29, 30).

### Protein Gel and Western Blot Analyses

Immunoprecipitated samples, purified recombinant human proteins (2 μg) or NHS (100 μg), were separated, either on a non-reducing 10% SDS-PAGE or on a reducing 12% SDS-PAGE with subsequent short colloidal Coomassie staining (31), or proteins were transferred onto polyvinylidene difluoride membranes. Membranes were blocked (3% BSA/PBS-T, 1 h) and subsequently incubated with biotinylated mAb RETC-2 (5–10 μg/mL, 3% BSA/PBS-T, 4°C, overnight). Membranes were treated with streptavidin-HRP (1:2500 in 3% BSA/PBS-T, 30 min) and developed with Lumi-Light blotting substrate (Roche Diagnostics GmbH, Mannheim, Germany).

### Sandwich-ELISA for Determination of FHR-3 Concentrations in Human Serum

MaxiSorp microtiter plates (Nalgene Nunc, Penfield, NY, USA) were coated with capture antibody mAb RETC-2 (10 μg/mL in PBS, overnight, 4°C) and blocked with 2% skim milk in PBS-T (1 h). Human serum samples (1:20, 1:40) and recombinant FHR-3 (1.4–1000 ng/μL), diluted in 2% skim milk in PBS-T, were incubated (1 h) for quantification. Followed by an incubation with biotin-labeled detection antibody mAb anti-FHR-3.4 (0.3 μg/mL) in 2% skim milk in PBS-T (1 h), detection of sandwich ELISA was performed with streptavidin-HRP (1:5000, 2% skim milk in PBS-T, 30 min) and TMB. Optical density was determined at 450 nm.

### Genetic Analysis

Genomic DNA was isolated from whole blood (P1, P2, N1, N2) (32). Amplification of the *cfhr3* gene and sequence analysis were performed using specific primers (R3) as described previously (14).

### Immunohistochemistry

Enucleated donor eyes fixed in 4% paraformaldehyde (48 h) were rinsed in PBS/0.05% azide, and the anterior segment was removed. The eyecups were cryo-protected in a 10–30% sucrose gradient (3 days) and embedded in frozen section medium Neg-50 (Thermo Fisher Scientific, Braunschweig, Germany). Immunofluorescence was performed on 25-μm thick sections. The slides were treated with blocking solution (PBS containing 3% DMSO, 0.3% Triton X-100, and 5% normal donkey serum) to reduce non-specific background (1 h). Primary antibodies [RETC-2, 60 μg/mL; rabbit anti-Iba1 polyclonal antibody, 1 μg/mL; goat anti-FH polyclonal antibody, 60 μg/mL; mouse anti-glutamine synthetase monoclonal antibody (mAb), 2 μg/mL] were incubated in blocking solution (overnight). Antibody binding was detected with secondary antibodies (goat anti-mouse CF488A-conjugate antibody, 1:1000; goat anti-rabbit Cy3-conjugate antibody, 1:500; rabbit anti-goat Alexa Fluor 546-conjugate antibody, 1:1000). Cell nuclei were stained with DAPI (1:1000). Images were taken with a custom-made VisiScope CSU-X1 Confocal System (Visitron Systems, Puchheim, Germany) equipped with a high-resolution sCMOS camera (PCO AG, Kehlheim, Germany).

### Real Time qRT-PCR

Primary RPE/choroid and retina were dissected from an unfixed human eye. Retinal cell populations were separated using magnetic-activated cell sorting (MACS) as described in Grosche et al. (33). Human RPE for cultivation was isolated from healthy donor eyes (age 86 and 65 years) and treated as previously described (34). ARPE19 cells were purchased from ATCC (LGC Standard GmbH, Wesel, Germany) and cultivated as reported earlier (35). Human liver cDNA was kindly provided by V. M. Milenkovic (Department of Psychiatry and Psychotherapy, University Regensburg). mRNA of the cells was isolated (NucleoSpin RNA/Protein Kit, Macherey-Nagel, Düren, Germany), and cDNA was synthesized (Quantitect Reverse Transcription Kit, Qiagen, Hilden, Germany). qRT-PCR was performed using Quantitect primer sets (*chfr3*: QT00001631, *cfh*: QT00001624, *gapdh*: QT00079247) and Rotor Gene Sybr green PCR Kit (Qiagen, Hilden, Germany). Taqman PCR was performed using Brilliant III UF MM QPCR/Low ROX master mix (Agilent Technologies, Waldbronn, Germany) and the following *cfhr3*-specific primer (*cfhr3*-forward: gtttgcaaaatggatggtca; *cfhr3*-reverse: ggaggtggtatcaccattgc) and the FAM-labeled probe #25 (Roche Diagnostics, Mannheim, Germany).

### ELISA for C3b Interaction

PolySorp microtiter plates (Nalgene Nunc, Penfield, NY, USA) were coated with C3b (10 μg/mL, PBS, overnight, 4°C). Blocking was performed with Casein Diluent Blocker (Senova GmbH, Weimar, Germany) (1 h). FHR-3 [10 μg/mL (200 nM)] and anti-FHR-3 mAb RETC-2 [100 μg/mL (666 nM)] were preincubated in PBS (1 h). Incubation of FHR-3, FH [2.6 μg/mL (16 nM)], and FHR-3 with anti-FHR-3 mAb RETC-2 on C3b plates (15 min) was performed. For the standard curves antigen serial dilutions (FHR3 0.7–1500 nM, FH 0.2–6451.6 nM, PBS) were incubated (1 h). Binding was detected either with mouse anti-FH mAb (2.5 μg/mL, PBS, 1 h) and anti-mouse IgG-HRP (1:5000 PBS, 30 min) or StrepMAB-HRP (1:40000, 30 min, PBS). The signal was developed with TMB, and the optical density (absorption) was determined at 450 nm.

### Sandwich ELISA for Heparin Interaction

MaxiSorp microtiter plates (Nalgene Nunc, Penfield, NY, USA) were coated with StrepMAB-classic (10 μg/mL in PBS, 4°C, overnight). After blocking (5% BSA/PBS-T, 1 h), recombinant human FHR-3 [250 μg/mL (5 μM)] with Strep-tag in PBS was incubated (1 h). Prior to heparin-biotin addition (100 μg/mL, 5% BSA/PBS-T, 1 h), anti-FHR-3 mAb or specific isotype control was incubated (8 mg/mL (53 μM), PBS, 1 h). Detection was followed by streptavidin-HRP (1:5000, PBS, 30 min) and TMB. The signal was measured at 450 nm.

### Software and Statistical Analyses

Immunogenic and unique peptides of SCR5 were determined by AbDesigner (36). Sequence analyses of mAb RETC-2 and mAb RETC-3 variable regions were performed with VBASE2 (37) and Rosetta online (38) server. The visualization of antibody structures and complementarity-determining regions (CDR) was realized with Chimera (39). Data were statistically analyzed using GraphPad Prism 5 (GraphPad Software, San Diego, CA, USA).

## Results

### Novel Mouse mAbs Specifically Interact with Human FHR-3

We generated four different mouse hybridoma cell lines against native human FHR-3 using a peptide immunization strategy. The isolated and purified four mAbs RETC-2, RETC-3, RETC-5, and RETC-7 were specific for the C-terminal, fifth SCR (SCR5) domain of FHR-3.

These antibodies were of different mouse isotypes. MAb RETC-3 and mAb RETC-7 were mouse IgG1 antibodies. MAb RETC-2 and mAb RETC-5 were mouse IgG2b isotypes (Table 1). κ-light chains complemented the FHR-3 binding structures of the variable heavy chain regions. Sequence analysis and *in silico* simulations revealed the specific, variable paratope structure (Fab), including the CDRs of mAb RETC-2 and mAb RETC-3 (Figure S1 in Supplementary Material).

**Table 1:** **Summary of anti-FHR-3 mAb and isotype controls**.

	RETC-2	RETC-5	IgG2b control	RETC-3	RETC-7	IgG1 control
**Species**	Mouse	Mouse	Mouse	Mouse	Mouse	Mouse

**Antigen**	FHR-3^a^	FHR-3^a^	Unknown	FHR-3^a^	FHR-3^a^	BSA

**Isotype**	IgG2b, κ	IgG2b, κ	IgG2b, κ	IgG1, κ	IgG1, κ	IgG1, κ

**ELISA**						

FHR-3	+	+	−	+	+	−

FHR-1	−	−	−	−	−	−

FHR-2	−	−	−	−	−	−

FHR-4A	−	n. d.	−	n. d.	n. d.	n. d.

FHR-4B	−	n. d.	−	n. d.	n. d.	n. d.

FHR-5	−	−	−	−	−	−

FH	−	−	−	−	−	−

BSA	−	−	−	−	−	+

NHS	+	+	−	+	n. d.	−

**Western blot**						

FHR-3	+	+	−	+	+	−

FHR-4A	−	n. d.	n. d.	n. d.	n. d.	n. d.

FHR-4B	−	n. d.	n. d.	n. d.	n. d.	n. d.

FH	−	−	−	−	−	−

BSA	−	−	−	−	−	+

NHS	+	+	−	+	n. d.	−

**FHR-3-specific IP**	+	n. d.	−	+	n. d.	−

**MAb-influence in protein interaction**						

C3b-FHR-3	↓	n. d.	n. d.	n. d.	n. d.	n. d.

C3b-FH/FHR-3-competition	↑	n. d.	n. d.	n. d.	n. d.	n. d.

Heparin-FHR-3	−	n. d.	n. d.	n. d.	n. d.	n. d.

*^a^SCR5 domain*.

*n. d., not determined*.

**Figure 1:**
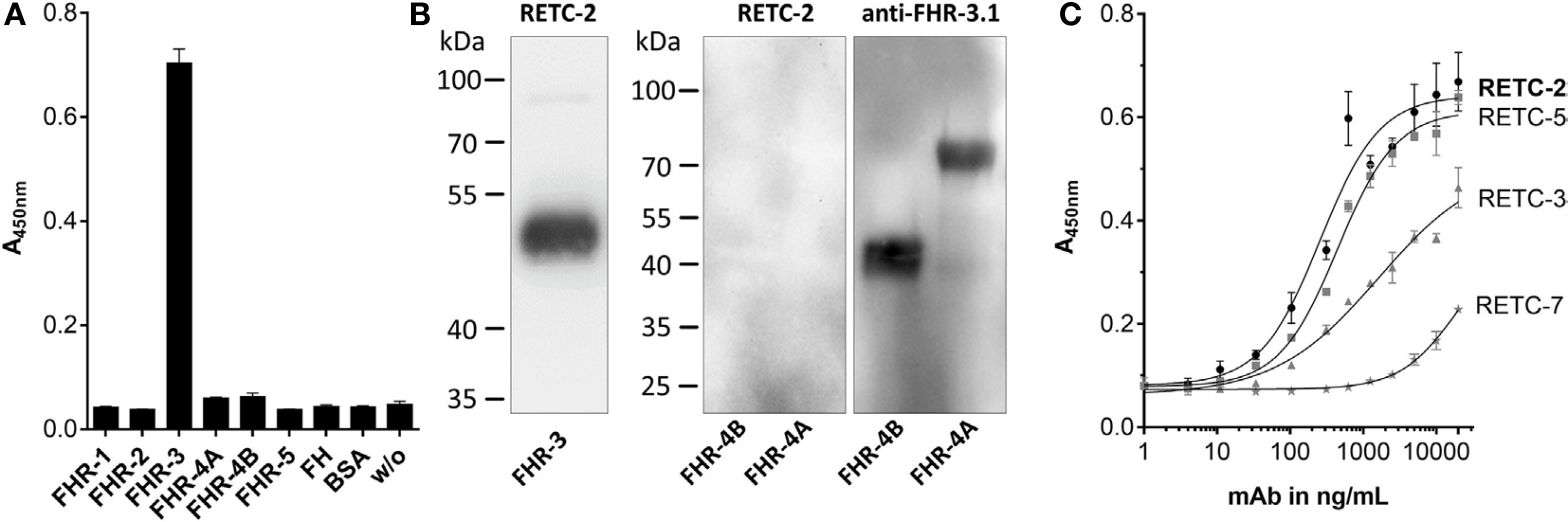
**Mouse mAbs were specific for recombinant human FHR-3**. **(A)** MAb RETC-2 reacted exclusively with FHR-3 but not with other proteins of the FH-family in ELISA. MAb RETC-3, RETC-5, and RETC-7 showed the same detection pattern (data not shown, see Table 1). **(B)** MAb RETC-2 revealed a specific detection of FHR-3 compared to the highly identical FHR-4A and FHR-4B (94% identity to FHR-3) in Western blot. Detection of recombinant proteins was performed either with mAb RETC-2 or with mAb anti-FHR-3.1. **(C)** MAbs RETC-2 and mAb RETC-5 (IgG2b mAbs) showed the highest binding signals for human, recombinant FHR-3 immobilized on microtiter plates.

Our analysis of the specificity of all four anti-FHR-3 mAbs disclosed significant detection of recombinant FHR-3 in ELISA and Western blots (Figures 1A,B; Table 1), neither FHs, related FHR-1, FHR-2, FHR-4A, FHR-4B, nor FHR-5 proteins, were bounded (Figures 1A,B; Table 1). All antibodies interacted with the same 11-amino acid long epitope in SCR5, albeit with different binding strengths (Figure 1C). The mouse IgG2b mAbs revealed the highest avidity to FHR-3, including RETC-2 (EC50 = 256 ng/mL) and RETC-5 (EC50 = 445 ng/mL), which differed by a factor of 1.7 (Figure 1C). The IgG1 mAbs RETC-3 (EC50 = 1617 ng/mL) and RETC-7 (EC50 = 19849 ng/mL) showed a 6–77 times lower binding strength to recombinant FHR-3 than the IgG2b mAbs (Figure 1C). Primarily, the antibody-target epitope interaction of mAb RETC-2 was characterized in-depth as this mAb showed the highest avidity against immobilized, recombinant FHR-3 (Figure 1C).

**Figure 2:**
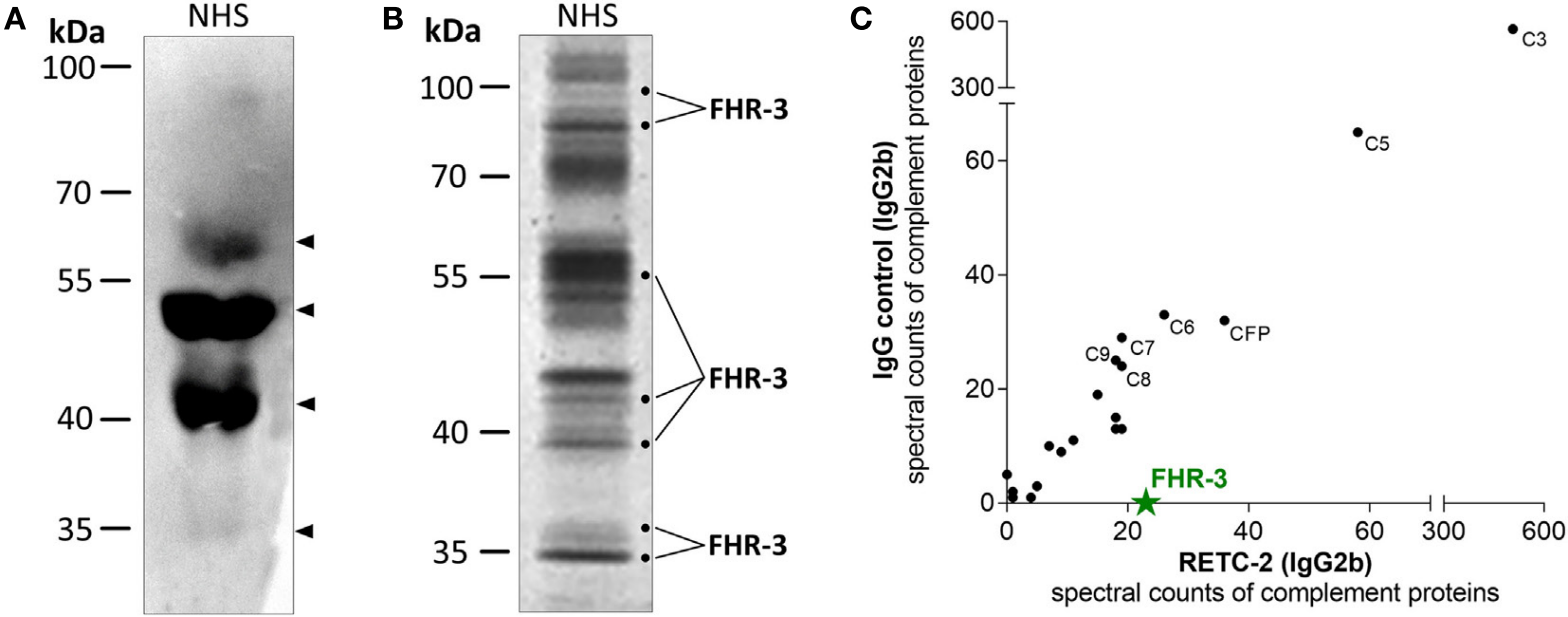
**MAb RETC-2 detected native FHR-3 in complex with other complement proteins from human serum**. **(A)** MAb RETC-2 detected four protein bands for FHR-3-glycoforms between 35 and 65 kDa (arrows) from reduced normal human serum (NHS) in Western blot. **(B)** More than 30 different proteins were immunoprecipitated from non-reduced NHS using RETC-2 and isotype control (not shown). Labeled fractions after Coomassie staining were identified by mass spectrometry analyses and contained FHR-3. Unlabeled protein bands contained other serum proteins (e.g., serum albumin, apolipoproteins, Igs). **(C)** RETC-2 specifically precipitated FHR-3 (green star) in comparison to an isotype control mAb. Additional peptide fragments, characterized with non-gel LC-MS/MS, were complement proteins.

We confirmed the specificity of anti-FHR-3 mAbs for FHR-3 produced naturally in the body by analyzing human blood samples in Western blots. FHR-3 was detected as a monomer in different FHR-3-glycoforms (between 35–65 kDa) in human serum without enrichment and under reducing conditions using anti-FHR-3-specific mAb RETC-2 (Figure 2A, arrows; Table 1). The glycoforms correspond to the previously described four glycosylation sites of FHR-3 (13, 40). MAb RETC-2 showed a detection ratio of 1:8:4:0.1 corresponding to the putative FHR-3 glycoforms at 65, 50, and 40 kDa, as well as a hardly detectable non-glycosylated form at 35 kDa (Figure 2A, arrows). Detection of FHR-3 using mAb RETC-2 from human serum under non-reducing conditions resulted in additional protein signals at approximately 90–100 kDa (Figure 2B).

Mouse Ig interacts with their variable Fab-part with the corresponding target epitope (Figure 1), and their constant Fc-part is recognized by Fc-receptors or complement components (41, 42). We used a combination of gel-based (Figure 2B) and non-gel LC-MS/MS (Figure 2C) protein identifications to dissect the interactions into Fab-part (anti-FHR-3 specific) and Fc-part (general mouse Ig isotype specific) mechanisms. Therefore, we compared the precipitated protein patterns from native human serum using either anti-FHR-3 mAb RETC-2 or the corresponding isotype Ig (Figures 2B,C; Table S1 in Supplementary Material). Fab-specific FHR-3 counts were exclusively identified in immunoprecipitations using the novel anti-FHR-3 mAb at different protein sizes (Figures 2B,C), but not with the isotype controls (Figure 2C; Table S1 in Supplementary Material). In contrast, FH and FHR-5 bound to all mouse IgG2b whereas FHR-1, FHR-2, or FHR-4 did not interact with the IgGs (Table S1 in supplementary material). Given the fact that no other FH-family members were consistently detected in combination with FHR-3, the data suggested that FHR-3 circulates *in vivo* as a monomer or as a homo-multimer.

The constant, non-specific Fc-antibody parts of both the anti-FHR-3 mAb and the isotype control mAb precipitated additional serum proteins (Table S1 in supplementary material). Separation of mAb RETC-2 precipitated proteins by SDS-PAGE with subsequent Coomassie staining revealed protein bands with 20 different mobilities (Figure 2B) and 10 bands with isotype control (data not shown). On average, 30 different proteins were identified as Fc- and/or immunoprecipitation-beads associated interaction partners for both the FHR-3 specific and the corresponding isotype control antibodies. These proteins included abundant proteins, such as keratin, apolipoproteins, Igs, and serum albumin. Our analysis focused on complement-associated proteins. More than 20 human complement proteins of all three complement activation pathways bound to the murine Fc-antibody parts IgG1 or IgG2b, irrespective of the antibodies specificity (Fab-part) (Figure 2C; Table S1 in Supplementary Material). The human complement proteins C3 and C5 were detected with the highest spectral counts as binding partners for all mouse Fc-parts (Figure 2C). MAbs RETC-2, RETC-3 as well as the corresponding isotype controls precipitated also proteins of the terminal pathway (C6–C9) and important soluble regulators (clusterin, vitronectin, properdin) (Table S1 in Supplementary Material). In addition, characteristic components of the classical pathway (C1, C2, C4), the lectin pathway (MASP1, ficolin-2), and the alternative pathway (CFB) were associated with mouse IgG2b but not with mouse IgG1 Fc-parts antibodies (Table S1 in Supplementary Material).

In conclusion, these results showed that the specific FHR-3-antibodies detected FHR-3 from human serum. However, we confirmed a general interaction of human complement proteins with the mouse Fc-antibody part. We concluded that mAb RETC-2 will need to be humanized in the future for further functional interaction studies involving this mAb and human serum containing all complement proteins.

### Quantified FHR-3 Levels Varied in Rheumatic Diseases and SLE

**Figure 3:**
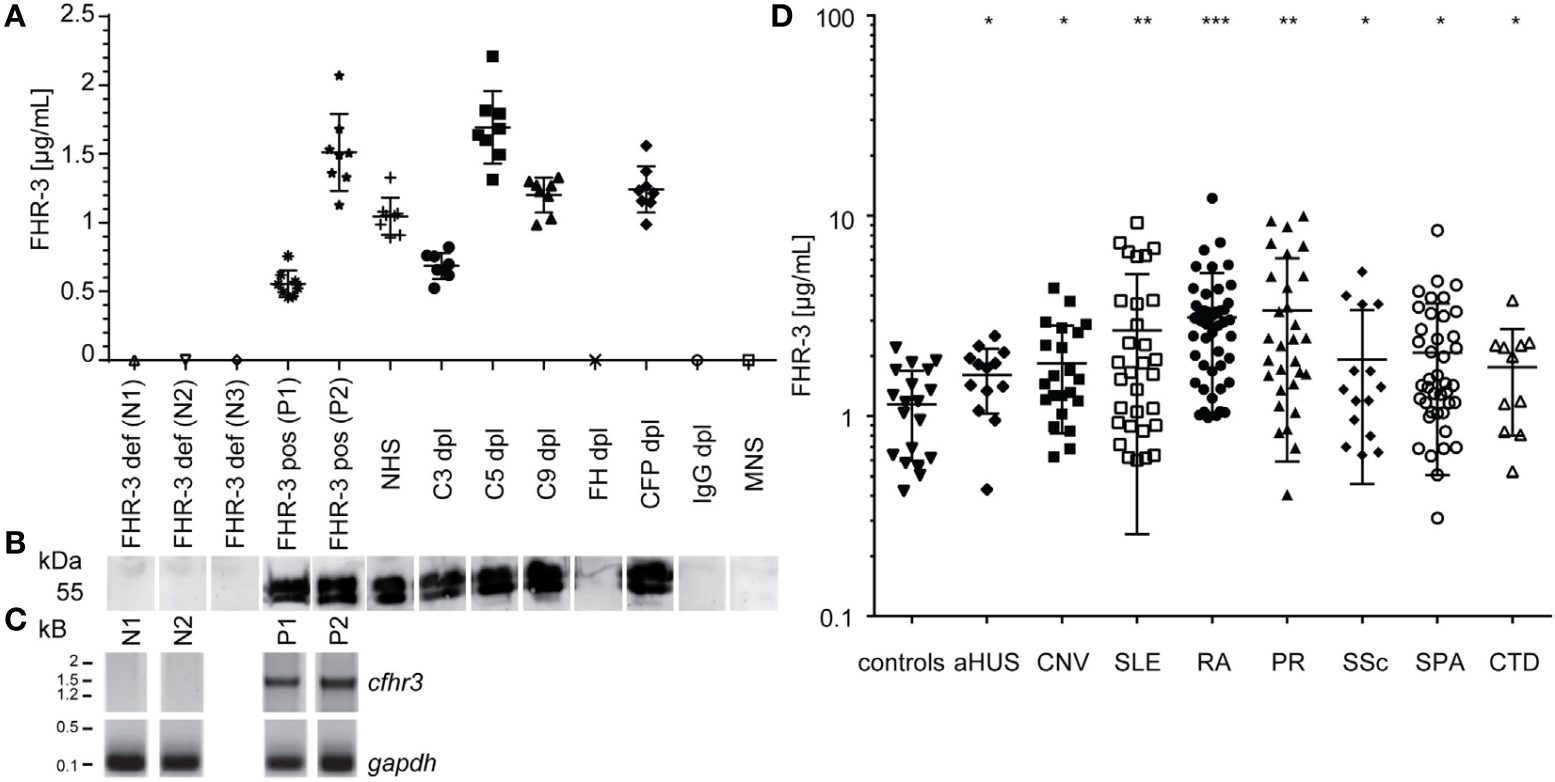
**FHR-3 quantification resulted in significantly increased FHR-3 serum concentrations for SLE, RA, and PR patients**. **(A)** Quantified FHR-3 serum levels, using a sandwich immunoassay showed a reproducible, specific detection pattern in concentration range between 0.5 and 1.7 μg/mL. Technical replicates from each sample are shown as calculated FHR-3 concentrations in μg/mL (standard curve not shown). **(B)** Immunoblots correlated with FHR-3 detection in the immunoassay and showed two bands between 50 and 55 kDa for specific FHR-3 immunoprecipitation from reduced serum samples. **(C)** *Cfhr3* genotyping was consistent with FHR-3 immunodetection. **(D)** Systemic lupus erythematosus (SLE), rheumatoid arthritis (RA), and polymyalgia rheumatica (PR) patients showed an increased FHR-3 concentration compared with healthy controls (Table S2 in Supplementary Material) in the immunoassay (aHUS, atypical hemolytic-uremic syndrome; CNV, choroidal neovascularization; SSc, systemic sclerosis; SPA, spondyloarthritis; CTD, connective tissue diseases). Means of two independent quantifications are shown. ***p < 0.001, **p < 0.01, *p < 0.1 (two-tailed, unpaired *t*-test).

An immunoassay for FHR-3 quantification from human samples was established, using mAb RETC-2 antibody as capture antibody and biotin-labeled anti-FHR-3.4 mAb (which reacts with FH and FHR-3) as detection antibody (6) (Figures 3A,D).

We performed chromosomal DNA analysis of leukocytes from healthy blood donors to determine positive and negative controls for the immunoassays. The positive samples (P1, P2) resulted in one specific 1.5 kb-fragment for the N-terminal *cfhr3*-gene region (SCR1/2), and negative samples (N1, N2) were homozygous for the *cfhr3* deletion (Figure 3C). FHR-3 serum levels from healthy positive (P1, P2) and negative (N1–N3) controls were consistent with the respective genotype and were detected without any false-negative or false-positive signals in the immunoassay (Figures 3A,C). The ELISA and genotyping results (Figures 3A,C) correlated with the immunoblot detection, showing protein bands at 50–55 kDa for FHR-3 positive serum samples and no signals for the FHR-3-deficient sera (Figure 3B).

Using this specific immunoassay, we aimed to determine FHR-3 levels in different standardized serum samples, using complement-deficient sera exemplarily (Figure 3A). Tested serum samples varied in their FHR-3 levels between 0.63 μg/mL for C3-depleted (C3^dpl^) and 1.69 μg/mL for C5^dpl^ sera (Figure 3A). FHR-3 was not detected in FH^dpl^, IgG^dpl^, or normal mouse serum (Figures 3A,B). This is explained by heparin-based immunodepletion of FH from the serum that also removed heparin-affine FHR-3.

Repetitive FHR-3 quantification using positive control and complement-depleted sera revealed an interassay variation of the sandwich ELISA of 12–23% (Figure 3A).

**Figure 4:**
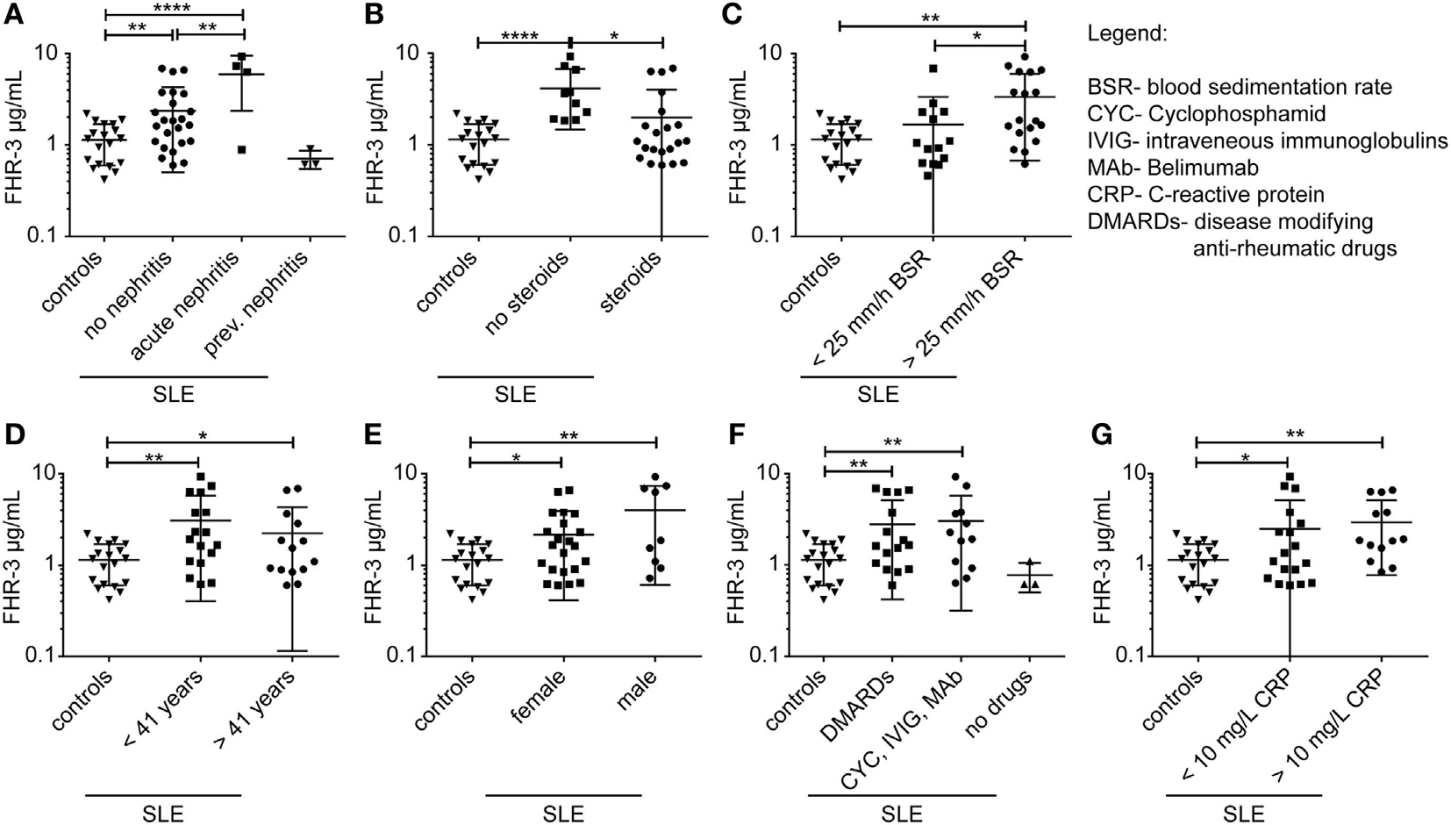
**FHR-3 serum levels in SLE patients correlated with lupus nephritis diagnose, steroid treatment, and blood sedimentation rate**. FHR-3 amounts in sera of SLE patients were determined in a FHR-3-specific immunoassay. We detected a significance difference in FHR-3 concentrations in patients **(A)** with acute lupus nephritis, **(B)** patients treated with steroids versus non-steroid treatment, and **(C)** patients with elevated blood sedimentation rates (BSR). However, **(D)** age, **(E)** sex, **(F)** medication, and **(G)** C-reactive protein (CRP) levels did not influence the systemic FHR-3 values in SLE patients in a biologically relevant manner. ***p < 0.001, **p < 0.001, *p < 0.1 (two-tailed, unpaired *t*-test).

We then investigated whether FHR-3 levels were systemically changed in complement-associated diseases (Figures 3D and 4; Table S2 in Supplementary Material). Serum samples from 21 healthy, young (mean age 26) volunteers showed a FHR-3 concentration of 0.41–2.49 μg/mL with a mean concentration of 1.06 + 0.53 μg/mL (Figure 3D; Table S2 in Supplementary Material), whereas the patient samples showed a FHR-3 concentration range of 0.31–12.29 μg/mL. On average, FHR-3 concentrations of patients with aHUS (mean 1.6 μg/mL), choroidal neovascularization (CNV, mean 1.83 μg/mL), systemic sclerosis (SSc, mean 1.92 μg/mL), and connective tissue diseases (CTD, mean 1.76 μg/mL) were slightly increased (Figure 3D; Table S2 in Supplementary Material). However, serum samples from non-steroid-treated SLE, rheumatoid arthritis (RA), and polymyalgia rheumatica (PR) patients were associated with potentially biological relevant, significantly increased FHR-3 serum levels (Figures 3D and 4; Table S2 in Supplementary Material). In our small cohort, the FHR-3 concentrations were threefold to fourfold higher in non-steroid-treated SLE (mean 4.14 μg/mL), RA (mean 3.12 μg/mL), and PR (mean 3.37 μg/mL) patients in comparison to controls (Figures 3D and 4B; Table S2 in Supplementary Material). Steroid treatment of SLE patients decreased the FHR-3 amounts in serum by 48% (Figure 4B). A correlation of FHR-3 levels and clinical parameters revealed a putative relation of FHR-3 levels and lupus nephritis diagnose or blood sedimentation rate (BSR) in SLE patients, respectively (Figures 4A,C). Increased C-reactive protein (CRP) levels in RA patients corresponded to higher FHR-3 concentrations (Table S3 in Supplementary Material). FHR-3-deficient serum samples were mainly found in aHUS (38%), non-steroid treated SLE (17%), and in control (14%) cohorts (Table S2 in Supplementary Material). FHR-3 protein analyses in the aHUS group were confirmed by genetic analysis of all samples using multiplex ligation-dependent probe amplification (data not shown).

In summary, these experiments showed that FHR-3 levels in serum were altered in complement-associated inflammatory diseases. This suggested that systemic FHR-3 concentrations were associated with complement activity and inflammation.

### FHR-3 Is Localized in Microglia/Macrophages and FH in Müller Cells in the Retina

**Figure 5:**
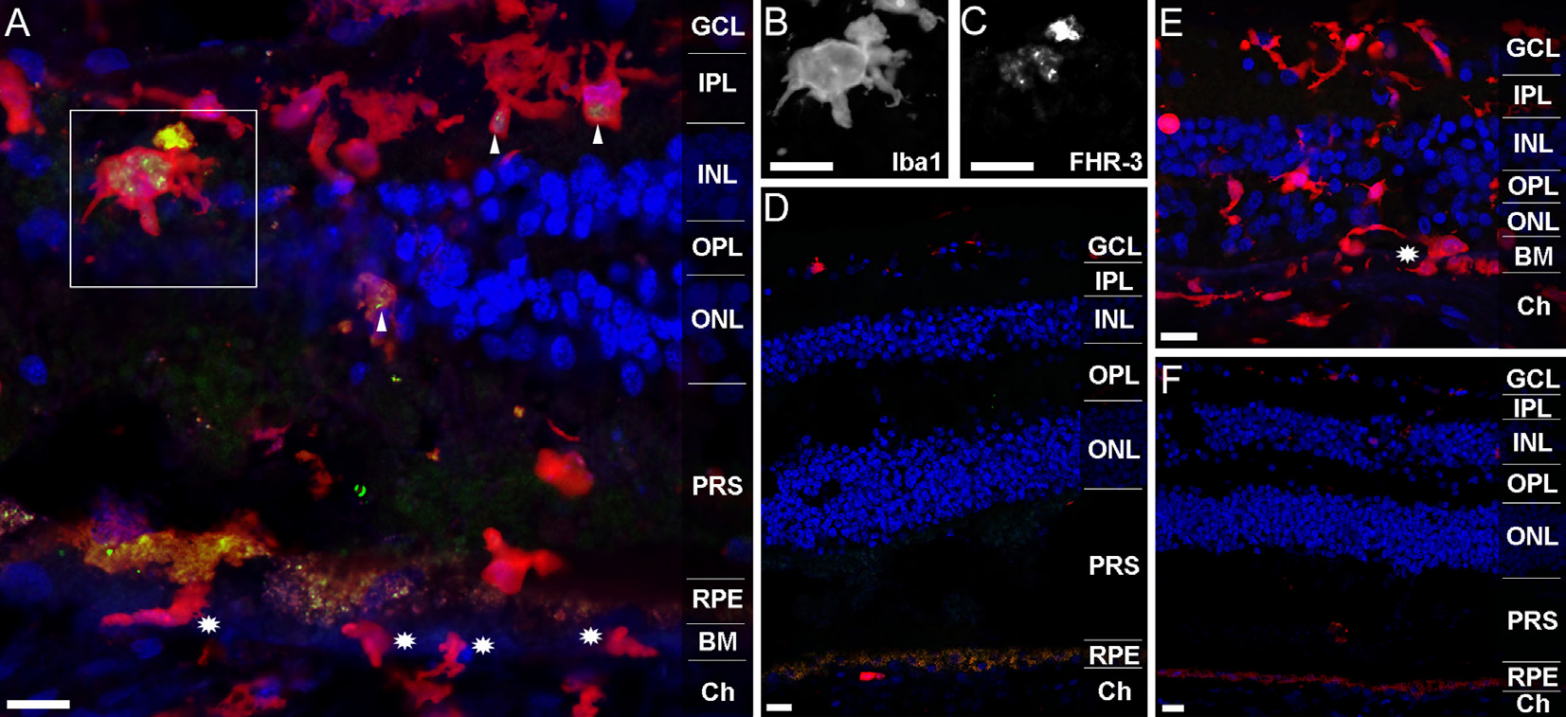
**FHR-3 is localized in human retinal microglia/macrophage cells**. FHR-3 (*green*) **(A,D)** was localized in microglia/macrophages (*red*) **(A,D,E)** in the central retina of the 92-year-old donor (*arrows*) **(A)**, but not in microglia/macrophages of a 64-year-old donor retina **(D)**. The marked area of **(A)** showing a microglia cell with an enlarged soma area, indicating microglia activation, is depicted in **(B,C)**. Note the partial **(A)** and complete **(E)** absence of autofluorescent RPE in the sections from the 92-year-old donor. *Stars* in **(A,E)** indicate invading macrophages and/or leaving activated microglia cells. **(E,F)** show an isotype control for anti-FHR-3, co-stained with anti-Iba1, and demonstrated the differences of microglia/macrophage distribution in **(E)** the retina of the aged and **(F)** the younger donor. Retinal layers from the top to the bottom: GCL, ganglion cell layer; IPL, inner plexiform layer; INL, inner nuclear layer; OPL, outer plexiform layer; ONL, outer nuclear layer; PRS, photoreceptor segments; RPE, retinal pigment epithelium; BM, Bruch’s membrane; Ch, choroid. Scale bars, 40 μm.

Previous studies revealed that FHR-3 deficiency is protective of AMD but a risk factor for the development of aHUS (9, 10, 13). We detected no critical differences in systemic FHR-3 protein concentrations of healthy donors compared with AMD or aHUS patients (Figure 3D). Therefore, a local effect of FHR-3 could be of relevance. We stained FHR-3 in a retina from a 92-year-old donor (Figure 5), which showed an increased number of invading macrophages (or activated microglia cells; Figures 5A,E) in comparison to a retina from a 64-year-old donor (Figures 5D,F). Surprisingly, we identified FHR-3 in activated microglia/macrophages in the central retina of the 92-year-old donor using mAb RETC-2 (Figure 5A, arrows; Figures 5B,C, magnified). FHR-3 was not detected in resting microglia/macrophage in the periphery of the younger retina (Figure 5D). Fluorescent signals in the RPE (Figures 5A,D,F) were identified as unspecific autofluorescence (Figure S2 in Supplementary Material). Interestingly, there is a patchy lag of autofluorescent RPE in the 92-year-old donor retina, indicative of local RPE atrophy. Invading macrophages and/or activated microglia were most prevalent in these tissue areas (Figures 5A,E). In contrast, the RPE appears to be largely intact in the 64-year-old control (Figures 5D,F).

In order to confirm the local retinal FHR-3 production, mRNA expression analysis from eye tissue was performed. We isolated mRNA from different RPE cells (primary human RPE cells, cultivated human RPE cells, and ARPE19 cells), as well as human liver cells as control, and tested *cfhr3* together with *cfh* expression using qRT-PCR (Figure S3 in Supplementary Material). *Cfhr3* mRNA was only detected in liver cells but not in RPE-cells. However, *cfh* specific mRNA was determined in RPE and liver cells (Figure S3 in Supplementary Material). Thus, different retinal cell types were separated from a human retina, and *cfhr3* expression levels were compared to *cfhr3*-mRNA in ARPE19 cells using Taqman PCR (Figure S3 in Supplementary Material). *Cfhr3* expression levels were 2- to 140-fold higher in microglia/macrophages isolated from human retina compared to other retinal cell types (Figure S3 in Supplementary Material).

**Figure 6:**
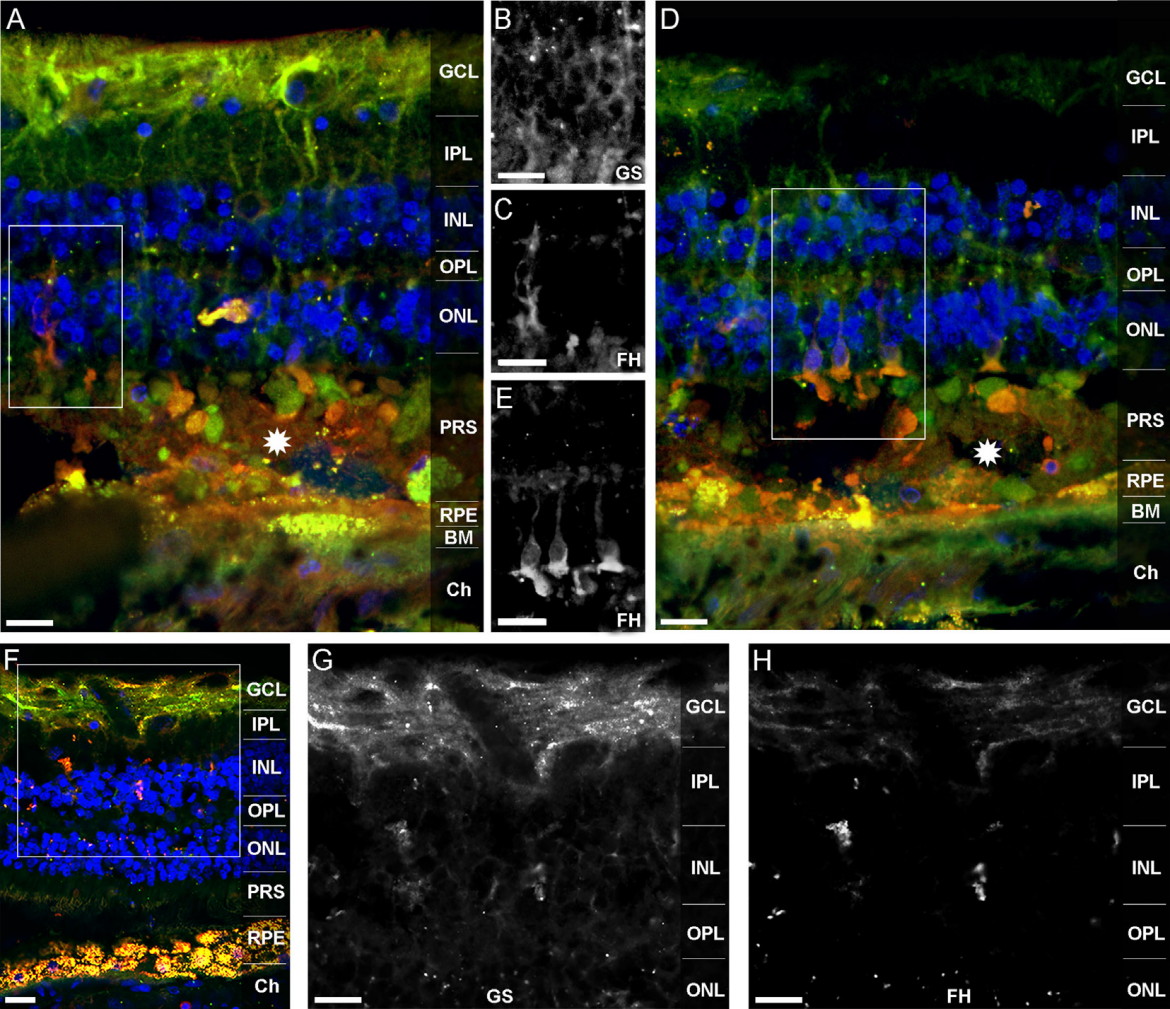
**FH is localized in human Müller cells and photoreceptor cell segments**. FH **(***orange* **A,D,F)** colocalized with different cellular sub-compartments of retinal Müller glia **(***green* **A,D,F)**. In the 92-year-old human retina, FH was stained in the outer stem processes of Müller cells, which is shown in the marked area of **(A)** and depicted in **(B,C)**. FH colocalized in the subretinal space of the 92-year old retina either with photoreceptor somata or with Müller cell processes enwrapping photoreceptor somata **(D,E)**. The marked area of **(D)** is shown in **(E)**. *Stars* in **(A,D)** indicate undefined subretinal debris. In the 64-year-old retina, FH was primarily present in the inner stem/perisynaptic processes of Müller cells and in Müller cell end feet **(F–H)**. The magnified, marked area of **(F)** is shown in **(G,H)**. Retinal layers from the top to the bottom: GCL, ganglion cell layer; IPL, inner plexiform layer; INL, inner nuclear layer; OPL, outer plexiform layer; ONL, outer nuclear layer; PRS, photoreceptor segments; RPE, retinal pigment epithelium; BM, Bruch’s membrane; Ch, choroid. GS, anti-glutamine synthetase mAb as Müller cell marker. Scale bars, 40 μm.

Factor H-related protein-3 is highly identical to FH and interacts with FH-binding partners. This suggested a similar binding pattern of FHR-3 and FH. Upon FH-staining of a human retina, Müller cells and photoreceptor cell segments were identified as FH-expressing cells (Figure 6). FH did not colocalize with FHR-3 in microglia/macrophages. The FH expression pattern in Müller cells appeared to be different in two investigated donor retinae. In the 92-year donor retina, the outer stem processes of Müller cells (Figures 6A–C), Müller cells enwrapping photoreceptor somata or the photoreceptor somata itself, and Müller cell microvilli reaching into the layer of photoreceptor segments were FH-positive (Figures 6D,E). In contrast, in the 64-year-old donor retina, FH labeling was more prominent in the inner stem processes (including perisynaptic side branches) of Müller glia spanning the inner plexiform layer (Figures 6D–F). This finding might be explained by the different degree of tissue damage as the disarranged retinal layers and activated microglia/macrophages in the 92-year-old retina were indicative of a more pronounced tissue inflammation with concomitant RPE loss and neurodegeneration compared to the 64-year-old donor (Figures 6A,D,F).

In summary, these stainings of the human retina and expression analyses demonstrated that FHR-3 expression might be locally regulated and that it is likely to depend on the activation level of microglia/macrophages. Our novel anti-FHR-3 mAb RETC-2 revealed that FH and FHR-3 were expressed in retinal cell types with entirely different functions.

### Anti-FHR-3 mAb RETC-2 Reduced Molecular Interaction of FHR-3 with C3b but Not with Heparin

Having shown that FHR-3 is expressed by microglia/macrophages in the retina (Figure 5), we asked whether our novel anti-FHR-3 mAb RETC-2, directed against the SCR5 domain, interferes with the local binding characteristics of FHR-3. Therefore, we used competitive immunoassays to analyze the effect of mAb RETC-2 on FHR-3 interactions.

**Figure 7:**
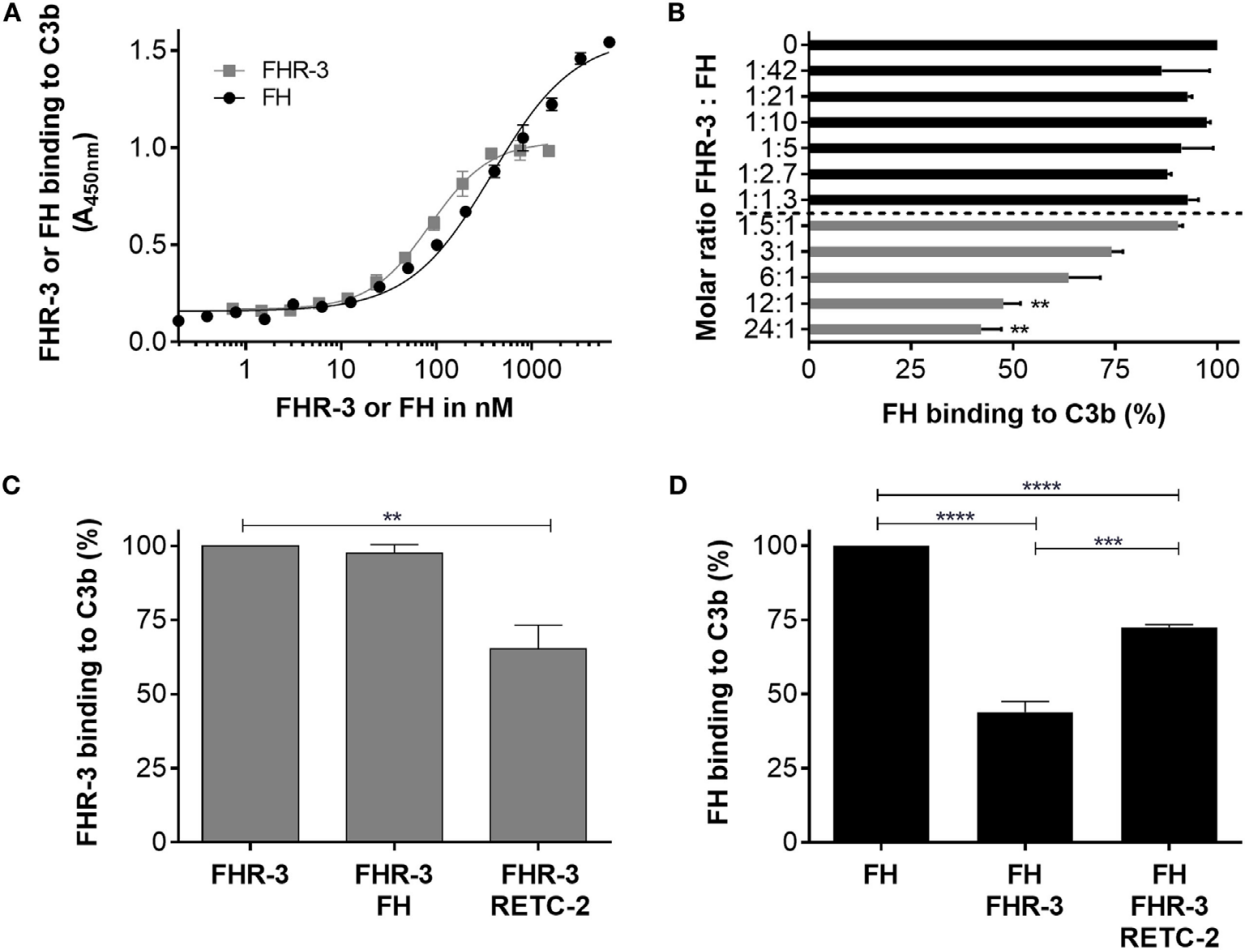
**Anti-FHR-3 mAb RETC-2 reduced binding of FHR-3 to C3b**. **(A)** FHR-3 and FH bind to C3b in a dose-dependent manner. **(B)** FH binding to C3b was reduced by FHR-3 in a dose-dependent manner. Statistic was performed using one-way ANOVA with multiple comparisons and FHR-3 (0 nM) as control (**p < 0.01). **(C)** MAb RETC-2, but not FH, reduced interaction of FHR-3 to immobilized C3b. Statistic was performed using one-way ANOVA with multiple comparisons and FHR-3 as control column (**p < 0.01). **(D)** FH binding to C3b was reduced by FHR-3. This inhibitory effect was reversed by the addition of mAb RETC-2. Data represent mean values +SD of three independent experiments. Statistics were performed using One-way ANOVA with multiple comparisons and FH as control column (****p < 0.0001) and two-tailed unpaired *t*-test (***p < 0.001).

Factor H-related protein-3 binds *in vivo* to C3b on cell surfaces mainly *via* a simultaneous interaction with glycosaminoglycan chains. C3b interacted *in vitro* (without glycosaminoglycan chains) with recombinant FHR-3 and native FH (Figure 7A). The FHR-3–C3b interaction was not influenced by the addition of FH (Figure 7C). However, when anti-FHR-3 mAb RETC-2 was added to FHR-3 and C3b, the FHR-3-C3b binding was reduced by 32% (Figure 7C). This indicated either that in addition to SCR5 further domains are involved in the interaction of FHR-3 with C3b or that the binding strength of mAb RETC-2 was not sufficient to replace FHR-3 completely.

In accordance with previous studies, we observed a competitive effect of FHR-3 and FH for C3b binding (5) (Figure 7B). A molecular ratio above 12 FHR-3 to 1 FH molecule significantly reduced the binding of FH to C3b (Figure 7B). MAb RETC-2 binding to FHR-3 ameliorated the FHR-3 interference with FH for C3b binding by 29% and resulted in an increased FH concentration bound to C3b (Figure 7D). We found stronger RETC-2 effects for the interaction of FH and FHR-3 with oxidative stress epitopes (manuscript in preparation).

Previous reports showed an *in vitro* interaction of recombinant FHR-3 with heparin (Figure S4 in Supplementary Material) (3, 43). In order to better understand FHR-3 interaction with cell surfaces, we investigated whether mAb RETC-2 interferes with FHR-3 binding to heparin, a model for highly sulfated glycosaminoglycan chains. We conclude that the FHR-3–heparin interaction was not influenced by anti-FHR-3 mAb RETC-2 (Figure S4 in Supplementary Material). The data indicate that the heparin-binding region in FHR-3 is not located in the SCR5 domain and thus not in the C-terminus of FHR-3.

These results showed that FHR-3 competes with FH for C3b binding and that this can be modulated by specific mAbs to the SCR5 domain of FHR-3 without affecting the interaction with heparin.

## Discussion

We here report of new, highly specific murine antibodies for a well-defined epitope of FHR-3, which we used for systemic as well as local detection and functional modulation of FHR-3. FHR-3 is a member of the FH protein family, which are important regulators of the complement system and associated with several diseases, such as AMD and aHUS (4). The specific detection of proteins depends mostly on distinct binding of antibodies to the target antigen. Due to the high sequence identities among the FH protein family, the majority of previously generated antibodies showed cross-reactivity with other FH-family members (Figure S5 in Supplementary Material) (6, 44), with the exception of mAb HSL1 (19). HSL1 binds to SCR4–5 of FHR-3 and was used to determine binding of FHR-3 to pathogens. However, a distinct binding epitope and functional modulation of FHR-3 using mAb HSL1 have not been described.

In this study, the anti-FHR-3 mAbs were generated against a selected peptide sequence of the SCR5 domain. The paralogous SCR20 in FH is known for most of disease-associated missense mutations (45–47) and binding of autoantibodies (48). This C-terminal domain of FH/FHR proteins also facilitates the binding to C3d and glycocalyx *in vivo* (49) (Figure S5 in Supplementary Material). Therefore, it was a favorable target for function-modulating antibodies.

Our mAb RETC-2 specifically detected FHR-3, native and denatured FHR-3, but no other FHRs. SCR9 domain of FHR-4A showed the highest identity with the FHR-3 target domain used for antibody generation (93.8%). Although only two amino acids (aa R285 and aa R288) of the immunogen were different in the corresponding region of FHR-4A, mAb RETC-2 was highly specific for FHR-3 and did not interact with FHR-4A. We exclusively precipitated FHR-3 and no other attached FH-family member from human serum, which supports the hypothesis that FHR-3 circulates as a monomer or homo-multimer in human serum, in contrast to FHR-1, FHR-2, and FHR-5, which form beside homo-multimers also heterodimers and share a dimerization motif (18).

Besides a previous estimation on FHR-3 levels (5), there has been only one other report using an immunoassay to measure FHR-3 levels in healthy individuals, reporting systemic FHR-3 levels of 0.7 μg/mL on average, and a major influence of copy number variation in *cfhr3* on the variation of the FHR-3 levels within a population (6). According to the high specificity of mAb RETC-2, we confirmed the FHR-3 concentration in human serum probes derived from healthy individuals in a concentration ranged between 0.41 and 2.49 μg/mL (excluding *cfhr3*-deficient donors). This concentration is lower than other soluble complement regulators in blood (e.g., 13–30 μg/mL properdin; 40–100 μg/mL clusterin) (24, 50) and even 9- to 1800-fold below the reported systemic FH concentration (116–562 μg/mL) (51).

Previous studies on the deletion of *cfhr3/cfhr1* genes revealed a protecting effect in AMD and IgAN but revealed a risk factor for aHUS and SLE (9, 10, 12, 13, 15). Serum FHR-3 concentrations in patients suffering from AMD or aHUS showed a similar FHR-3 concentration range as healthy controls, although there seemed to be a trend toward higher levels in this small cohort. These results should be further investigated in larger patient cohorts. However, in probes from patients with the autoimmune diseases SLE, RA, and PR threefold to fourfold increased systemic FHR-3 levels were detected.

Systemic lupus erythematosus is a complex disease with heterogeneous sub-phenotypes, which are all characterized by the production of autoantibodies, complement dysregulation, and inflammatory tissue injury (52). In previous studies, reduced FH levels and FH deficiency have been associated with SLE (53, 54). This indicates that the FH-homeostasis is highly relevant and that FH-competitors, such as FHR-3 (5), could affect disease progression. While we did find significant differences in FHR-3 levels within our SLE cohort, our results only include few patient samples and further studies are required to elucidate the role of FHR-3 in different clinical SLE phenotypes.

Additionally, we tested patients suffering from autoimmune diseases for FHR-3 levels, as we assumed elevated systemic FHR-3 levels could be involved in inflammation (16). Complement activation is important in RA as immune complexes fix complement, resulting in the release of chemo-attractants for macrophages (55). It is known that FH is an important inhibitor of this complement activation in joints (56). FHR-3 has not been found to be involved in RA pathogenesis so far, but mouse studies recently revealed a higher mRNA concentration of mouse CFHR-C in the spleen of RA mice compared to healthy mice (57). It remains unclear whether increased systemic FHR-3 concentrations could result in local competition of FH and FHR-3 in joints and result in complement dysregulation.

Polymyalgia rheumatica is associated with inflammation in proximal joints (58) and viral stimulation of the immune system, including autoantibodies and classical pathway activation, has been proposed as a possible pathomechanism (59, 60). Almost 30 years ago, Smith et al. identified proteins of the FH-family in immune complexes of PR patients (61). According to the specificity of the used anti-FH antibody at that time, the detected proteins could correspond to FHR-2, FHR-3, FHR-4, FHR-5, or FH-like than to the described FH (1). An association of FHR-3 with PR has not been described before, but increased systemic FHR-3 levels support the hypothesis of complement dysregulation in PR.

Concluding that systemic FHR-3 levels were not exceedingly altered in AMD patients, we focused further on the local presence of FHR-3 to decipher the role of *cfhr3/cfhr1* gene deletion in this disease. FHR-3 and members of the FH-family are primarily known as secreted plasma proteins, which are produced in liver cells (40). In addition, a myeloma cell line was also tested positive for *cfhr3* transcripts (62). Here, we describe the local expression of *cfhr3*/FHR-3 on mRNA and protein levels in the retina by putatively activated microglia or invading macrophages. Active microglia/macrophages are a common characteristic in retinal degenerative diseases and are associated with the release of inflammatory complement components (63–65). Increased local FHR-3 concentration in the damaged retina could influence the FH/FHR-3 balance. However, FHR-3 did not colocalize with FH in the human retina, as the FH protein was found in Müller and photoreceptor cell segments. So far, FH was detected at the choroidal site of the Bruch’s membrane and in RPE in the human retina (66). The previously reported FH-expressing RPE forms the blood-retina barrier and the Müller cells span the whole retina (66). Both cell types are known for complement protein expression, suggesting a responsibility for maintenance of the immune homeostasis in the eye (67–69).

The local binding and competition of FHR-3 and FH on surfaces is an important mechanism for the regulation of immune homeostasis (5, 19). We showed that 12 times more FHR-3 than FH molecules are needed for the displacement of FH on immobilized C3b *in vitro*. Systemic physiological conditions are associated with a FHR-3:FH ratio of 1:33 to 1:7400 (70). This relationship could be altered in pathophysiological processes. Reduced FH concentrations in stressed RPE and the detection of FHR-3 produced by macrophages in the damaged retina suggested a local shift of the FHR-3/FH equilibrium in retinal degeneration (71, 72).

The interaction of mAb RETC-2 with FHR-3 partly influenced the competition of FHR-3 and FH for binding to C3b. As mAb RETC-2 binds in SCR5 of FHR-3, this suggests that SCR4 and SCR5 (19) of FHR-3 are involved in C3b binding (Figure S5 in Supplementary Material). Indeed, the relevant amino acids in SCR19 of FH involved in C3b binding are conserved in SCR4 and SCR5 of FHR-3 (22, 73) (Figure S5 in Supplementary Material). Our results are in agreement with previous studies, which showed that both C-terminal domains of FHR-3 (SCR 4–5) mediated the binding of FHR-3 to C3b, indicating that SCR5 is involved but not sufficient for C3b binding (3).

Factor H-related protein-3 interacts with cell surfaces not only *via* its C3b/C3d but also *via* its heparin-binding sites (3). MAb RETC-2 did not interfere with heparin binding to FHR-3, suggesting that the heparin-binding site is not located in SCR5 of FHR-3. These results are supported by the fact that the relevant amino acids for heparin binding identified in SCR20 of FH are not conserved in SCR5 of FHR-3 (Figure S5 in Supplementary Material). Instead, a high amino acid identity is present between SCR2 of FHR-3 and SCR7 of FH, the latter including a heparin-binding site (1). Thus, FHR-3 probably binds with its N-terminal domain and not *via* the C-terminus to heparin (Figure S5 in Supplementary Material).

**Figure 8:**
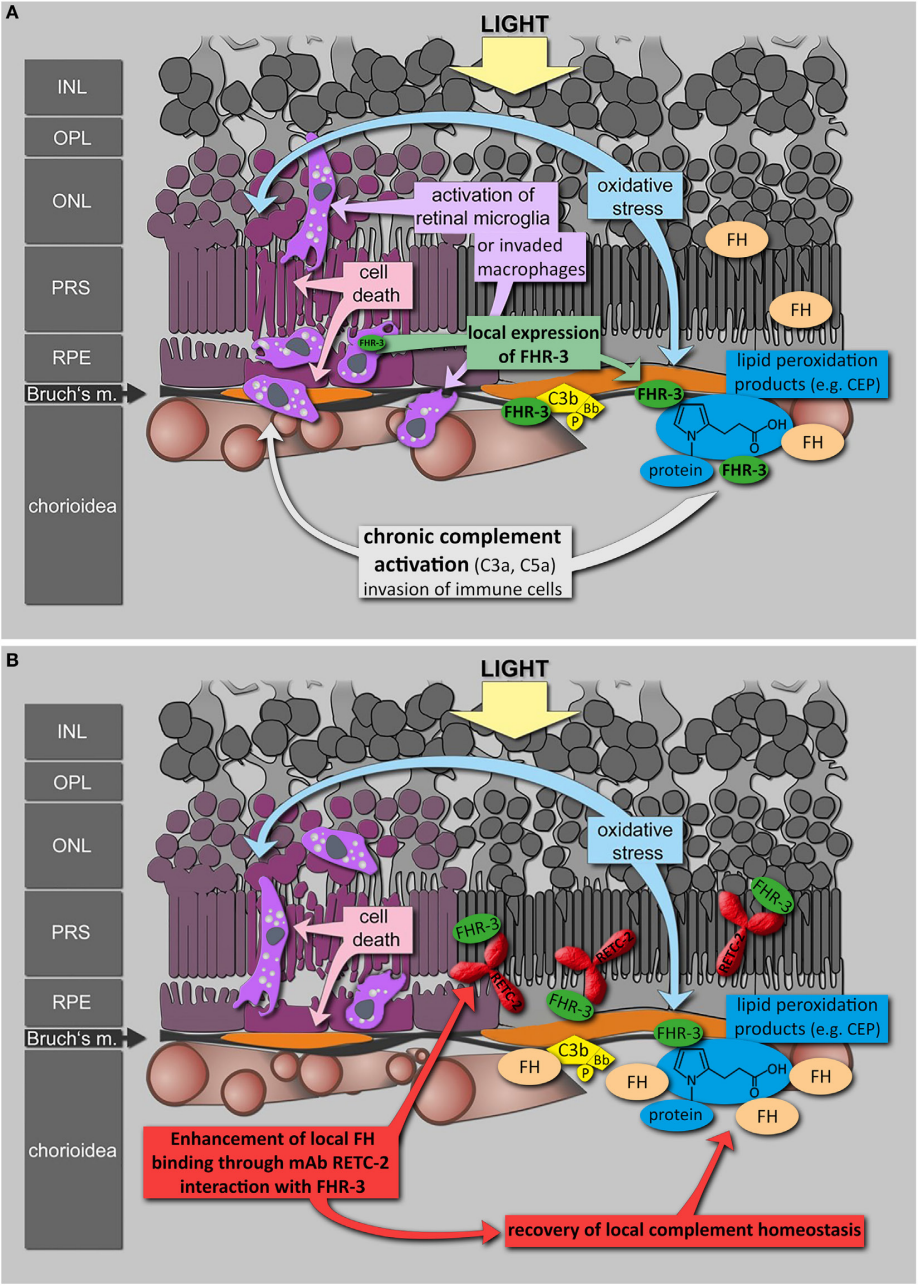
**Putative role of FHR-3 in retinal degeneration and the therapeutic potential of mAb RETC-2**. **(A)** FHR-3 is locally produced in the retina by microglia cells/invading macrophages and competes with FH for binding to C3b and modified surfaces. FHR-3 disturbs the FH-homeostasis in the retina. It potentially modulates complement activation following oxidative stress at the RPE/choroid and leads to progression of retinal degeneration. **(B)** MAb RETC-2 inhibits FHR-3 interaction with surfaces, lipid peroxidation products, and enhances binding of FH to C3b in the eye. This could result in a recovery of local complement homeostasis and a reduced progression of retinal degeneration.

To date, antibodies targeting the immune system offer promising therapies in different autoimmune diseases (74–76). Based on genetic association studies, FHR-3 seems to be a Janus-faced target: beneficial effects needed in aHUS and deleterious effects avoided in AMD. Inhibition of FHR-3 may therefore confer a therapeutic option in some autoimmune pathologies. Given the lack of effective therapy for the dry form of AMD and the reported local FHR-3 production in the damaged eye, FHR-3 inhibition by a humanized version of RETC-2 may be an effective therapeutic strategy (Figure 8). An unbalanced, local FHR-3/FH equilibrium could be the cause for local activation of microglia/macrophages in the human retina, as FH can not efficiently bind to the modified cell surfaces (Figure 8A). MAb RETC-2 interfered with FHR-3 binding to C3b fragments and enhanced FH binding. Our FHR-3 specific mAb may have a positive effect in the retina, helping to restore the local homeostasis by enhanced FH binding on cell surfaces to neutralize stress reactions (Figure 8B). Additional investigations of the systemic role of FHR-3 in RA, SLE, and PR are necessary to approve a potential therapeutic effect of FHR-3 blockade in systemic autoimmune diseases.

## Conclusion

In summary, we generated specific antibodies against FHR-3, which detected endogenous FHR-3 in the retina and allowed to determine FHR-3 serum concentrations. Enhanced serum levels of FHR-3 found in sera from patients with autoimmune diseases, such as RA, SLE, and PR, underline the important role of FHR-3 in homeostasis. Our study confirms that FHR-3 competes with FH for C3b binding *via* SCR5 and binds to heparin *via* the N-terminal region. As the novel RETC-2 antibody inhibits competition of FHR-3 with FH and thus enhances local FH binding, our newly generated mAb RETC-2 may be a useful therapeutic target in specific autoimmune diseases.

## Author Contributions

NS and DP developed concept and designed the study. NS, AG, and DP designed experiments. NS, AG, JR, SH, VE, and DP performed experiments. NS, AG, BE, SH, VE, CS, and DP analyzed and discussed data. RP, TK, DW, BE, VE, PZ, and CS provided material. NS, AG, PZ, CS, and DP wrote the manuscript. RP, TK, and DW discussed and commented on the manuscript.

## Conflict of Interest Statement

NS, AG, JR, SH, RP, TK, DW, BE, VE, CS, and DP declare that the research was conducted in the absence of any commercial or financial relationships that could be construed as a potential conflict of interest. PZ received speaker honorarium from Alexion Pharmaceuticals.

## Abbreviations

aHUS, atypical hemolytic-uremic syndrome; AMD, age-related macular degeneration; BSA, bovine serum albumin; BSR, blood sedimentation rate; CDR, complementarity-determining regions; CNV, choroidal neovascularization; CRP, C-reactive protein; CTD, connective tissue diseases; CYC, cyclophosphamide; DAPI, 4′,6-diamidino-2-phenylindole; DMARDs, disease-modifying antirheumatic drugs; ELISA, enzyme-linked immunosorbent assay; Fab, variable antigen binding part of an immunoglobulin; Fc, constant part of an immunoglobulin; FH, complement factor H; FHR, FH-related protein; HRP, horseradish peroxidase; Ig, immunoglobulin; IgAN, IgA nephropathy; IVIG, intravenous immunoglobulins; mAb, monoclonal antibody; MNS, mouse normal serum; NHS, normal human serum; pAb, polyclonal antibody; PR, polymyalgia rheumatica; RA, rheumatoid arthritis; RETC, regensburg therapy complement; SCR, short consensus repeat domain; SLE, systemic lupus erythematosus; SPA, spondyloarthritis; SSc, systemic sclerosis; TMB, 3,3′,5,5′-tetramethylbenzidine.
