# Supplementary material for: Complement Regulator FHR-3 Is Elevated either Locally or Systemically in a Selection of Autoimmune Diseases
Source: Front Immunol. 2016 Nov 28;7:542. doi: 10.3389/fimmu.2016.00542 (PMC5124756; doi:10.3389/fimmu.2016.00542)
Supplement: Supplementary file 1 [file Data_Sheet_1.DOCX]

Supplementary Material

**COMPLEMENT REGULATOR FHR-3 IS ELEVATED EITHER LOCALLY OR SYSTEMICALLY IN A SELECTION OF AUTOIMMUNE DISEASES**

**Nicole Schäfer^1^, Antje Grosche^2^, Joerg Reinders^3^, Stefanie M. Hauck^4^, Richard B. Pouw^5,6^, Taco W. Kuijpers^6,7^, Diana Wouters^5^, Boris Ehrenstein^8^, Volker Enzmann^9^, Peter F. Zipfel^10,11^, Christine Skerka^10^, Diana Pauly^1*^**

*** Correspondence:** Diana Pauly, diana.pauly@ukr.de

# Supplementary Data

## Supplementary Figures


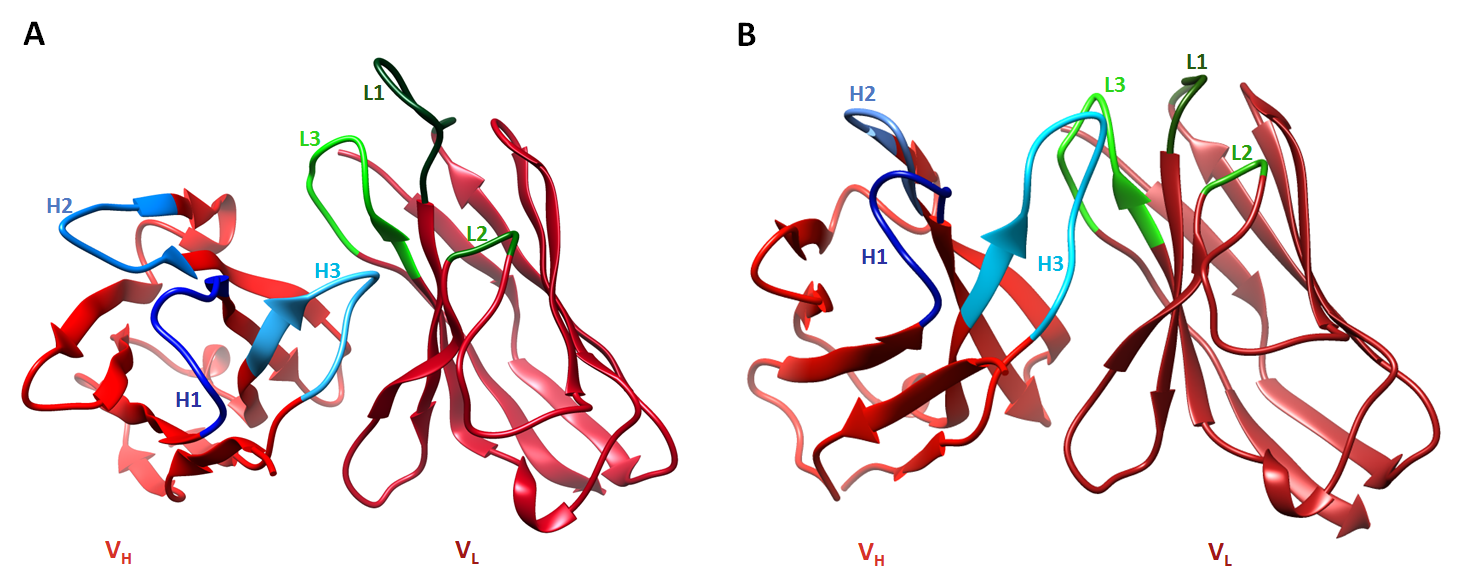


**S1 Fig. Structure of FHR-3-binding regions of mAbs.**

An *in silico* modelling of the antigen-binding regions of **(A)** mAb RETC-2 and **(B)** mAb RETC-3 show the variable heavy (V_H_, left) and light (V_L_, right) chain. The binding cleft is determined by six CDR loops (blue H1–H3 for the heavy chains and green L1–L3 for the light chains). Modeling was performed with Chimera.


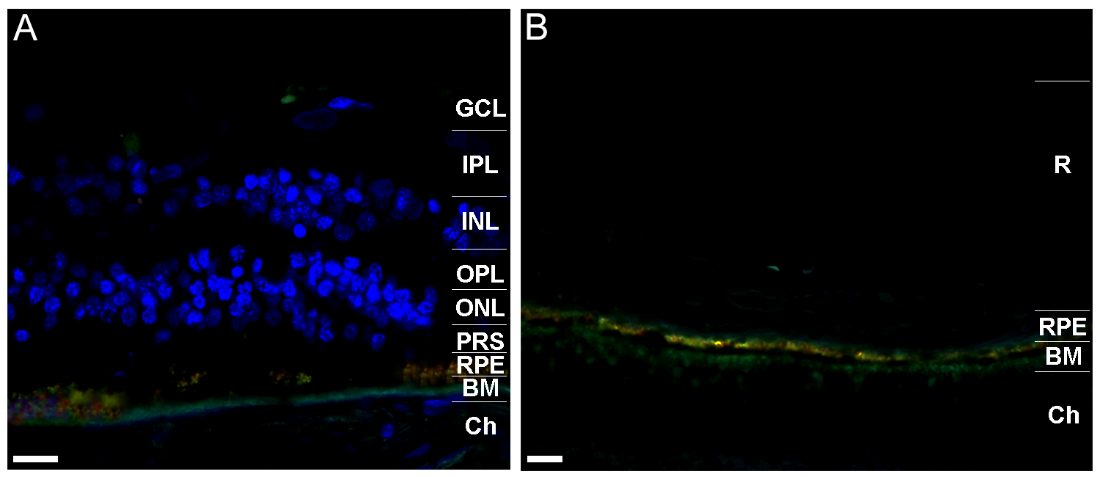


S2 Fig. RPE and Bruch`s membrane show autofluorescence in the human retina.

Autofluorescence of RPE (yellow) and Bruch’s membrane (green) in cryosections of a 92-year old retina are shown. Slices were either incubated **(A)** with secondary antibodies (w/o primary antibody) or **(B)** w/o antibodies**.**

Retinal layers from the top to the bottom: GCL, ganglion cell layer; IPL, inner plexiform layer; INL, inner nuclear layer; OPL, outer plexiform layer; ONL, outer nuclear layer; PRS, photoreceptor segments; RPE, retinal pigment epithelium; BM, Bruch’s membrane; Ch, choroid; R, retinal layers. Scale bars, 40 µm.


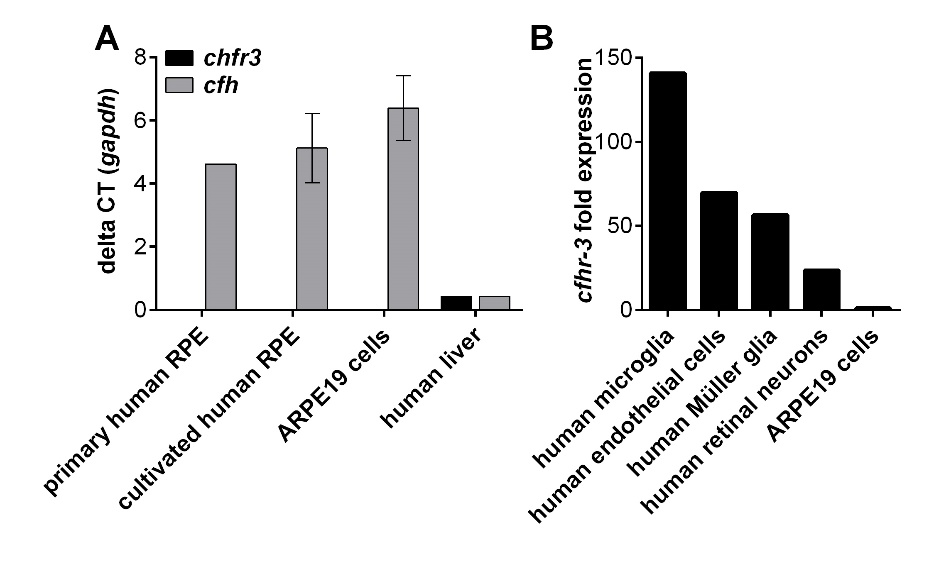


S3 Fig. *Cfhr3* mRNA is detected in microglia/macrophages but not in RPE cells

**(A)** *Cfhr3* was exclusively detected in liver cells. *Cfh* was expressed in RPE and liver cells. mRNA of human RPE different origins and liver cells was isolated. Expression of *chfr3* and *cfh* was analyzed using quantiTect primer in a two-step qRT-PCR. **(B)** *Chfr3* was mainly expressed in microglia/macrophages. mRNA of separated cell types from a human retina was isolated. *Chfr3* expression was analyzed with Taqman-PCR.





**S4 Fig. MAb RETC-2 has no effect on FHR-3 binding to heparin.**

**(A)** Soluble heparin-biotin binds to immobilized FHR-3 in a concentration dependent manner. Dotted line represents background signal of immobilized FHR-3 and detection reagent Streptavidin-HRP without heparin-biotin. **(B)** MAb RETC-2 did not reduce the binding of heparin to FHR-3. Data represent mean values ± standard deviations of two independent experiments.


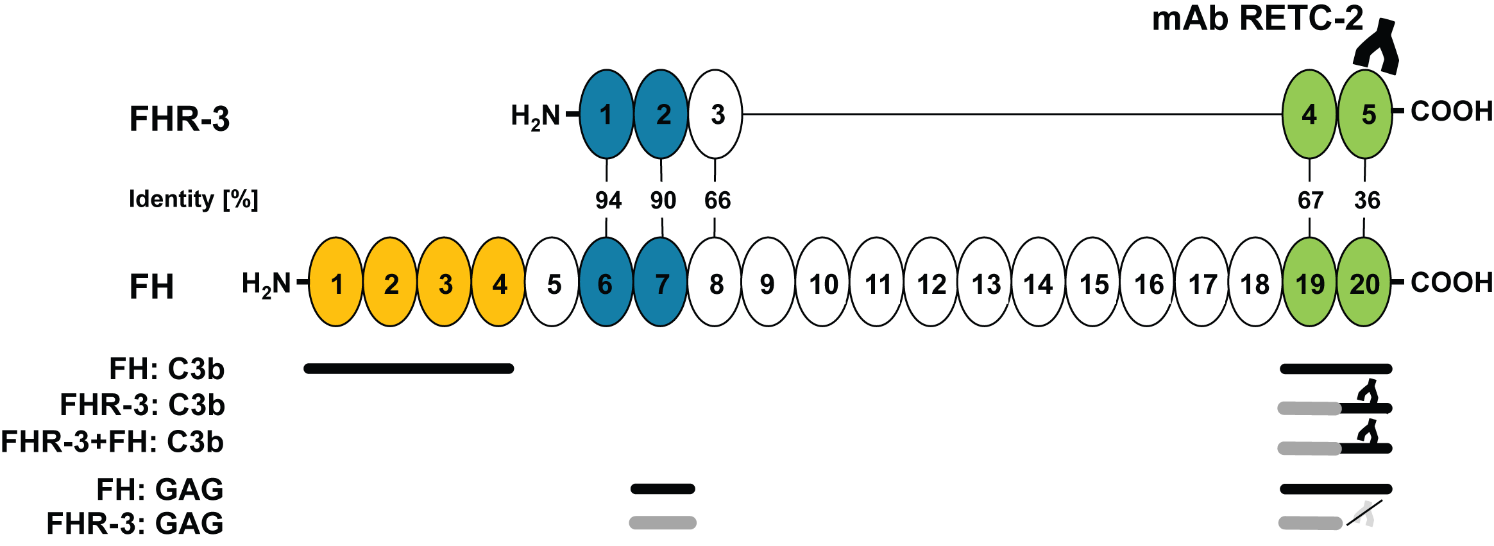


**S5 Fig: Anti-FHR-3 mAb RETC-2 modulates FHR-3 binding.**

MAb RETC-2 binds to SCR5 of FHR-3. SCR domains of FHR-3 (SCR1–5) and FH (SCR1–20) are depicted and aligned according the amino acid identities. The main recognition sites of FH are shown in black. Putative corresponding FHR-3 recognition sites are displayed in grey. Evidenced relevant FHR-3 binding sites using mAb RETC-2 are shown in black with antibody (modified after (P. F. Zipfel and Skerka 1994)).

## Supplementary Tables

**S1 Table** Identification of precipitated complement proteins from human serum using anti-FHR-3 mAbs and isotype controls

| Proteomics approach | Gel LC-MS/MS-analysis | | Non-Gel LC-MS/MS-analysis | | | |
| --- | --- | --- | --- | --- | --- | --- |
| Precipitated complement proteins | RETC-2 (IgG2b) | Isotype control (IgG2b) | RETC-2 (IgG2b) | Isotype control (IgG2b) | RETC-3 (IgG1) | Isotype control (IgG1) |
| FHR-3 | + |  | + |  | + |  |
| FHR-5 | + | + | + | + |  |  |
| C1q | + | + | + | + | + | + |
| C1s | + | + | + | + |  |  |
| C1r | + | + | + | + |  |  |
| C2 | + |  | + | + | + |  |
| C3 | + | + | + | + | + | + |
| C4bBP | + | + | + | + |  |  |
| C4 | + | + | + | + | + | + |
| C5 | + | + | + | + | + | + |
| C6 | + | + | + | + | + | + |
| C7 | + | + | + | + | + | + |
| C8 | + | + | + | + | + | + |
| C9 | + | + | + | + | + | + |
| FH | + | + | + | + |  |  |
| CFB | + | + | + | + |  |  |
| Ficolin-2 |  | + | + | + |  |  |
| MASP1 |  | + |  |  |  |  |
| Vitronectin | + | + | + | + | + | + |
| Clusterin | + | + | + | + | + | + |
| Properdin | + | + | + | + | + | + |
| CR1 | + |  |  |  |  |  |
| CR1L | + |  |  |  |  |  |

**S2 Table** Serum sample cohort

| Group | Disease | No | FHR-3^-/-^ (No.) | Age | | FHR-3 [µg/mL] | |
| --- | --- | --- | --- | --- | --- | --- | --- |
|  |  |  |  | range | mean | range | mean |
| controls | healthy, young | 21 | 14% (3) | 21–46 | 26 | 0.41–2.49 | 1.06+0.53 |
| aHUS | atypical uremic syndrome | 21 | 38% (8) | 11–82 | 34 | 0.43–2.52 | 1.60+0.57 |
| CNV | choroidal neovascularization | 22 | - | 64–88 | 77 | 0.63–4.36 | 1.83+1.0 |
| SLE  all  untreated | systemic lupus erythematosus | 33  12 | 6% (2)  17% (2) | 19–76  18–76­­ | 42  44 | 0.60–9.24  1.84–9.24 | 2.68+2.42  4.14+2.65 |
| RA | rheumatoid arthritis | 46 | - | 27–85 | 60 | 0.98–12.29 | 3.12+2.07 |
| PR | polymyalgia rheumatica | 30 | 3% (1) | 41–86 | 69 | 0.41–9.99 | 3.37+2.77 |
| SSc | systemic sclerosis | 16 | 6% (1) | 23–85 | 60 | 0.64–5.26 | 1.92+1.46 |
| SPA | spondylarthritis | 41 | - | 20–62 | 39 | 0.31–8.46 | 2.08+1.57 |
| CTD | connective tissue disease | 11 | - | 26–69 | 49 | 0.53–3.79 | 1.76+0.96 |

**S3 Table** Pearson correlation coefficient of FHR-3 levels and clinical parameters

|  | SLE | RA | PR | SSc | SPA | CTD |
| --- | --- | --- | --- | --- | --- | --- |
| Age | -0.148 | -0.022 | 0.130 | 0.238 | -0.018 | 0.471 |
| Sex | **-0.350*** | 0.224 | -0.050 | 0.070 | -0.047 | 0.104 |
| CRP | 0.289 | **0.247*** | 0.188 | -0.023 | 0.209 | **0.660*** |
| BSR | 0.264 | 0.021 | 0.187 | 0.133 | -0.071 | **0.777**** |
| Steroids | -0.395 | - | - | - | - | - |

*** p<0.001, ** p<0.01, *p<0.1 (two-tailed, pearson correlation).

Note: CTD cohort (n=11) includes very different disease manifestations
